# Nefopam prescribing preferences in French hospitals: results of a survey

**DOI:** 10.11604/pamj.2022.41.213.33365

**Published:** 2022-03-15

**Authors:** Thomas Schulz, Laure Lalande, Frederic Aubrun, Mikhail Dziadzko

**Affiliations:** 1 Département d’Anesthésie-Réanimation, Hôpital de la Croix-Rousse, Hospices Civils de Lyon, Université Claude Bernard, F-69004 Lyon, France,; 2 Service de Pharmacie, Hôpital de la Croix-Rousse, Hospices Civils de Lyon, Université Claude Bernard, F-69004 Lyon, France,; 3 Consultation Douleur, Groupement Hospitalier Nord, Hospices Civils de Lyon, F-69004 Lyon, France,; 4 Research on Healthcare Performance Lab, Université Claude Bernard, F-69008 Lyon, France

**Keywords:** Hospital physicians, nefopam, off label use, oral administration, prescribing pattern, regulation, survey

## Abstract

**Introduction:**

nefopam is a non-opioid, centrally-acting analgesic, frequently prescribed in France for acute pain and postoperatively. Only intravenous (IV) formulation is available, however the off-label oral use is frequent in surgical and medical patients. There is no data on the actual in-hospital prescription preferences in French physicians regarding nefopam. We wish to identify nefopam prescription habits for acute and chronic pain among hospital physicians.

**Methods:**

an online survey was sent to physicians via professional emails. Frequency of prescription, indication, preferred and prescribed administration route, dose regimen, and personal perception of the nefopam tolerance and efficiency were examined.

**Results:**

a total of 527 responses were analysed. Nefopam was mostly prescribed by senior hospital physicians, for acute pain, orally (85%), 20 mg/6h with 120 mg maximal daily dose. For chronic pain, the oral administration was more frequent. More than half of prescribers considered the efficacy of the oral route was similar to intravenous, and better tolerated compared to intravenous administration. Forty-eight percent of responders would change their prescription attitude in case of oral route approval of nefopam.

**Conclusion:**

oral prescription of intravenous formulation of nefopam is frequent, especially for acute pain, and has the same dose and regimen pattern as for intravenous route. Prescribers consider oral nefopam efficient and safe for patients. Regulatory actions regarding the oral nefopam prescription authorization and duration of such prescription are needed.

## Introduction

Nefopam hydrochloride is a tricyclic compound derived from diphenhydramine and developed in the early 1970s firstly as an antidepressant [1]. This is a non-opioid, centrally acting drug with a profile distinct from that of anti-inflammatory drugs, inhibiting the reuptake of serotonin, norepinephrine and dopamine [2]. Nefopam has been shown to possess analgesic and antihyperalgesic properties [3]. In France, it is widely used as perioperative co-analgesic for multimodal analgesia, but also in chronic pain such as migraine, facial pain and lumbosciatica, hence off label [4-7]. The recommended daily dose of intravenous nefopam in a non-comorbid adult is 20 mg every 4 hours (120 mg daily) for a maximum of 28 days. Intravenous formulation of nefopam is the only one available in France, however the off-label oral use is frequent (up to 5/1 000 adults in general population), both in community and inpatient settings [7, 8]. Such practice is in sustained increase since 2002 despite the absence of solid scientific evidence for the oral use [9].

In the context of surgery, nefopam is used as a part of multimodal analgesia not only perioperatively, but also after the patient´s discharge from the hospital [10]. Off-label in-hospital orders are frequent, where intravenous nefopam is prescribed as *“néfopam - ampoule pour préparation injectable - versé sur un morceau de sucre” - “nefopam - vial for injection - give on a piece of sugar”* [7], both in surgical and medical patients for acute pain (Annex 1 - Section 1).

In case of outpatient surgery, particularly orthopaedics - one of the most common functional procedures, nefopam is commonly used as complementary postoperative analgesic. It is prescribed at hospital before discharge, with continuous injection of 120 mg of nefopam using an elastomeric pump at the patient´s home, or as a single intravenous perfusion in the evening after discharge, made by visiting nurses [11, 12]. The oral use of intravenous formulation is also possible, however there is no data on such practices. Restrictive French regulation regarding strong opioids prescriptions and withdrawal of dextropropoxyphene (weak opioid, widely prescribed in France before 2011) from the market, undoubtedly contributed to the growth of nefopam use as a single analgesic or in combination with other pain relievers [13].

Despite the increasing prescription rate of intravenous formulation of nefopam given orally, there are no guidelines to formalize such attitude. Frequent off-label use leads to heterogeneous practices between physicians, however, there are no data available on physicians´ attitude concerning doses, regimen, duration, security and safety of oral prescription of this intravenous formulation. The aim of this study was to identify nefopam prescription preference patterns for acute and chronic pain management in hospital physicians.

## Methods

The study was exempted from the Ethical approval committee of our institution (Hospices Civils de Lyon); as no patients were involved, no written consent was necessary from the participating practitioners.

For the purpose of our study, we developed an online anonymous survey containing 12 items (Annex 1 Section 2). Data collected were: practice type, professional status, age, frequency of prescription of nefopam, indication for prescription, preferred (single choice) and prescribed (multiple choice questions) administration route, dose regimen prescribed intravenously and orally, and personal perception of the nefopam tolerance and efficiency (Likert-type questions). It was an anonymous online questionnaire generated using SoSci Survey tool [14] and it was available to responders between January 2019 and March 2019.

The questionnaire was sent via professional e-mail to all the physicians of the Hospices Civils de Lyon (second-largest university hospital group in France, Auvergne-Rhône-Alpes region), to all participants of the regional congress of anesthesiology and intensive care medicine (ICAR, 2019 edition, Auvergne-Rhône-Alpes region), and to physicians-members of the French Society for Pain Evaluation and Management (SFETD). Inclusion criteria were the responses from physician holding hospital position, including residents, and from those who prescribe nefopam. Excluded were incomplete questionnaires.

Data were recorded and processed using Microsoft® Excel® 2016 (16.0.2566.1000). Descriptive statistics were used, data was presented as frequencies and percentage. No sample size calculation was performed for this survey, because we were not able to find any relevant information on the expected prescription rate of nefopam among in-hospital physicians. If we consider the prescription rate at 50%, and margin of precision at 5%, we would need to analyse at least 385 responses to obtain a statistical power of 80% with 95% confidence level [15].

A comparative analysis was performed using χ^2^ or Fisher test as appropriate. The Marascuilo procedure was used to compare proportions between groups, where a significant χ^2^ test statistic was observed. The significance threshold was set at p<0.05.

## Results

A total of 531 questionnaires were collected within three months. We excluded 4 questionnaires (three were incomplete; and one reported no nefopam use), finally 527 responses were analysed. The response rate was 13.6%, based on the number of e-mail addresses used (3872 unique e-mail addresses were sent).

The majority of responders (86.7% n=457) were from public structures - university hospitals (59.5%) and general hospitals (27.3%); ten percent were from private hospitals, and about 3% from the mixed hospital-community practice. Seventy-two percent were senior physicians. Twenty percent (105 responders) reported daily prescription of nefopam, and more than 50% (301) prescribed nefopam regularly. Only 3% (16) of responders prescribed nefopam in an exceptional manner.

Hospital practitioners prescribe nefopam mostly for acute pain (55% for acute surgical pain and 47% for non-surgical acute pain), chronic pain was reported by 30% of physicians as a reason for prescription.

Prescribers declared that their preferred route of administration was the oral route (47%), followed by intravenous (29% drip perfusion and 22% continuous perfusion), isolated intramuscular (0.9%), and subcutaneous (0.7%) routes. In their current practice, 68% (n=355) responders prescribed nefopam using more than one administration route. The oral route was chosen in 85% of cases, followed by the intravenous route (62% for drip perfusion and 43% for continuous administration). Intramuscular (3%) and subcutaneous (5%) administrations were used occasionally.

**Figure 1:**
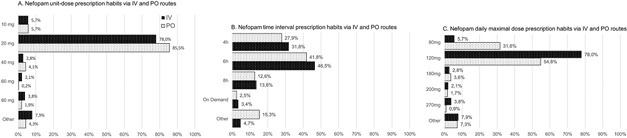
prescribing patterns for oral and intravenous nefopam

For the oral route, practitioners advised to ingest nefopam on a piece of sugar (75%), diluted in water (18%), or non-diluted (8%). Ten percent of responders had no preferences regarding the modality of oral administration. Figure 1 illustrates nefopam prescription patterns in unit dose (A), time interval between prescribed doses (B), and maximal daily dose (C) for intravenous and oral routes (Figure 1).

The most prescribed unit dose was 20 mg, both orally and intravenously. We did not find significant differences between prescribed unit doses according to the practice location (teaching hospital (p=0.47), general hospital (p=0.85), private hospital or community (p=0.10) or the preferential indication (acute post-operative pain (p=0.27), acute medical pain (p=0.20) or chronic pain (p=0.22)).

Six hours interval followed by 4 hours interval were the most common interval between nefopam administrations. A significant difference was found when comparing responders who used nefopam for chronic pain to other responders (Khi^2^=66.7; p<0.0001), with the 6h interval being less used for chronic pain (32.2% vs 47.6% p=0,031).

The distribution of prescribed maximal daily dose was significantly different when comparing the different modalities of practice, with the independent practitioners (office based) considering 120 mg the maximal dose less often than others (16.6%. Vs 54.3% p<0,0001). For chronic pain prescriptions, the preferred route was the oral route (94%), with unit doses of 20 mg every 4 (38%) to 6 (41%) hours.

Most responders considered oral nefopam as effective as intravenous nefopam (56.2%), 17.5% did not pronounce on this statement, while 26.3% did not think oral nefopam was as effective as IV nefopam. Most responders thought that oral nefopam was better tolerated than IV nefopam (53.2%), 16.4% did not pronounce on this statement and 30.4% did not think so. Almost all practitioners (91%) were aware of the bitter taste of nefopam, and less than 10% considered the taste as being neutral. Finally, 48.2% of responders estimated that marketing authorization for an oral formulation of nefopam would change their practice.

## Discussion

We report nefopam prescription habits of more than 500 French hospital-based practitioners. Nefopam was prescribed mostly by senior physicians from hospitals, mostly for acute pain, preferentially by oral route on a piece of sugar, using a 20 mg unit dose every 6 hours with a maximal daily dose of 120 mg. For chronic pain, the reported frequency of administration was greater, however the maximal daily dose remained the same. More than half prescribers considered oral route being as efficient as intravenous, and was better tolerated. 48% of responders would change their prescription attitude if an oral route of nefopam was approved.

The current French regulation authorised only parental route for nefopam with a recommended regimen of 20 mg every 4-6 hours and maximal cumulated dose of 120 mg over 24 hours. These doses were mainly reported by responders in our study but for the oral use, therefore off-label. Available studies on the efficacy of orally given nefopam are rare, and do not meet contemporary methodology [16, 17]. One randomized study targeting oral administration of intravenous formulation of nefopam for postoperative pain in the settings of orthopaedic surgery is ongoing [18].

Given orally, intravenous form of nefopam is first metabolized in the liver, its bioavailability is about 36±13% [19]. The maximal plasma concentration is achieved in 90-120 min [19]. Hence, intravenous formulation of nefopam given orally may need dosing regimen adjustments with increased unit dose, but such adjustments were only reported by a minority of responders to our survey. In countries where oral nefopam is approved, recommended doses are: 30 to 60 mg given 3 times a day (Belgium), and 60 to 90 mg given 3 times a day (United Kingdom).

Most responders believed that orally given nefopam was as efficient as intravenous, even if the administered dose was the same (20 mg). There are few studies comparing pharmacokinetics of oral vs intravenous equivalent doses of nefopam [19, 20]. The presence of an active metabolite, N-desmethyl-nefopam, which appears after the first-pass liver metabolism, may contribute to the analgesic effect of single dose nefopam solution administered orally. When the effect of desmethyl-nefopam is taken into account, the bioavailability may be considered as 62 ±23% [19]. We did not ask physicians to give a reason for their beliefs regarding the efficiency of the orally given intravenous formulation. The empirical practice, experience and examples from other intravenous medications given orally (e.g. - oral administration of intravenous solution of midazolam in pediatric and geriatric patients) may contribute to such opinions.

There is a growing use of nefopam in France, with 132% increase reported between 2007 and 2012 [21]. The French Agency for the Safety of Health Products (ANSM) reported “frequent” oral administration of intravenous formulation even before 2002 [7]. Along with acetaminophen, nefopam is the only non-opioid and non-anti-inflammatory agent analgesic available in France [13, 22]. Synergistic effect of orally administered nefopam and paracetamol on surgical pain relief was demonstrated recently in an animal model [23], and is being tested in a phase III multicentre study for the office based surgery [24]. Non-available in France, an oral formulation of nefopam (30 mg tablets) exists in several European countries including Belgium, United Kingdom and Germany, however no data is available on its use in ambulatory surgery.

The tolerance data for oral nefopam are scarce. Acute hypersensibility, psychiatric disorders, cutaneous manifestations were more frequent after intravenous administration, rather than oral [7, 25]. Nefopam can cause seizures and has antimuscarinic side effects (urinary retention, dizziness, blurred vision, dry mouth, tachycardia). Nefopam is contra-indicated in patients with a history of convulsive disorders and is not recommended for the elderly. Addiction potential of intravenous nefopam is believed to be more important in cases of long term administration. The drug dependence profile of nefopam is believed to be close to that of a psychostimulant. A recent French study reported a moderate risk of addiction to nefopam [26]. Addicted patients were more likely to receive nefopam via parenteral route and to suffer from chronic pain. Moreover, the analysis of data on the reimbursement of nefopam in the general population showed that one French person out of two having a prescription for nefopam, presented with chronic pain, which implies a long term use of this medication.

The interest of nefopam for management of chronic pain was already demonstrated [27, 28]. Unfortunately, we were not able to identify trials of orally given nefopam for chronic pain. In countries possessing oral formulation of nefopam, the only approved indication is acute pain [29]. Despite the lack of data, our survey showed a frequent use of the oral route in chronic pain. Given the widespread empirical oral use of intravenous formulation of nefopam, there is urgent need for an appropriate regulation, as well as clinical studies focusing on clinical effects in patients with persistent and chronic pain. In patients discharged after ambulatory functional surgery, the oral nefopam may play an important role as co-analgesic within the first 48 hours post-discharge, where the pain control is not optimal in more than 20% of patients [30].

Nefopam has a unique place among non-opioid analgesics because of its mechanism of action, and may be combined with other pain-relievers from the first step (acetaminophen, metamizol and non-steroid anti-inflammatory drugs) and 2^nd^, 3^rd^ step (weak and strong opioids) of the World Health Organization (WHO) analgesic ladder [31]. The absence of an appropriate oral formulation, the gap in regulation regarding oral use of intravenous form, and the established practice based on empirical experience may expose both patients and physicians to risk of errors, malpractice and abuse.

Limitations: our study has several limitations. First, there was a low response rate. However, some researchers have shown that lower response rate may provide more accurate measurements compared to surveys with higher response rates [32]. Then, selection bias may be present, especially since the majority of responders were from public hospital structures. Only 3% of responders were mixed -office based health practitioners, who are theoretically more prone to use the oral route of prescriptions. Prescription patterns in outpatient settings are largely understudied, however 86.1% of nefopam prescribers in France are general practitioners [26]. Finally, this study was based on declarative information. The tendencies observed here are though consistent with French reimbursement data [26]. To our knowledge, this survey is the first to evaluate the habits and the opinions of hospital-based physicians regarding nefopam administration.

## Conclusion

In-hospital oral prescription of intravenous formulation of nefopam in France is frequent, both for acute surgical (47%), non-surgical (55%) and chronic pain (94%). Despite the evidence of significantly low bioavailability of oral nefopam, responders used the same doses and regimen as for intravenous route. More than a half of prescribers considered intravenous formulation of nefopam efficient when given orally, and reported the need for an official approval of the oral use. Regulatory actions regarding the oral nefopam prescription authorization and duration of such prescription are needed, as well as well-designed randomized studies of the effect of oral nefopam on acute postoperative and chronic pain.

### What is known about this topic

- Nefopam, a non-opioid analgesic, is widely used in France, however only intravenous formulation is available;
- In the current in-hospital practice an off-label administration route - oral - is frequent, however there is no national recommendation on this topic.

### What this study adds

- More than 500 in-hospital physicians report their habits to prescribe nefopam, regardless clinical situation, patient speciality or professional category;
- In almost half of cases intravenous nefopam is prescribed orally - e.g. off label - in the hospital, without taking in account bioavailability;
- We illustrate an important problem of the misuse of intravenous formulation of nefopam, widely prescribed orally, because of the absence of regulation.

## Annex

**Annex 1**: a screenshot from the hospital health record-automated order and a survey used for the study

## References

[1] Klohs MW, Draper MD, Petracek FJ, Ginzel KH, Ré ON (1972). Benzoxazocines: a new chemical class of centrally acting skeletal muscle relaxants. Arzneimittelforschung.

[2] Sanga M, Banach J, Ledvina A, Modi NB, Mittur A (2016). Pharmacokinetics, metabolism, and excretion of nefopam, a dual reuptake inhibitor in healthy male volunteers. Xenobiotica.

[3] Chae JW, Kang DH, Li Y, Kim SH, Lee HG, Choi JI (2020). Antinociceptive effects of nefopam modulating serotonergic, adrenergic, and glutamatergic neurotransmission in the spinal cord. Neurosci Lett.

[4] SFAR Committees on Pain and Local Regional Anaesthesia and on Standards (2009). Expert panel guidelines 2008. Postoperative pain management in adults and children. Ann Fr Anesth Réanimation.

[5] Beloeil H, Albaladejo P, Sion A, Durand M, Martinez V, Lasocki S (2019). Multicentre, prospective, double-blind, randomised controlled clinical trial comparing different non-opioid analgesic combinations with morphine for postoperative analgesia: the OCTOPUS study. Br J Anaesth.

[6] Kim KH, Abdi S (2014). Rediscovery of nefopam for the treatment of neuropathic pain. Korean J Pain.

[7] Haut Autorité de Santé (HAS) (2021). COMMISSION DE LA TRANSPARENCE Avis 9 novembre 2016: néfopam (chlorhydrate de). Accesed on 26^th^ February.

[8] Bouvenot G, Juillet Y, Saint-Pierre A, Serre M-P (2018). Les Prescriptions médicamenteuses hors AMM (Autorisation de Mise sur le Marché) en France Une clarification est indispensable. Bull Académie Natl Médecine.

[9] Compte-rendu de la réunion de la Commission nationale des stupéfiants et des psychotropes du 21 juin 2012.

[10] Badaoui R, Rebibo L, Thiel V, Perret C, Popov I, Dhahri A (2014). [Observational study on outpatient sleeve gastrectomy]. Ann Fr Anesth Reanim.

[11] Garnaud B, Mares O, L´hermite J, Vialles N, Gricourt Y, Lannelongue A (2021). Multimodal oral analgesia strategy after ambulatory arthroscopic shoulder surgery: case series using adaptive therapeutic approaches by sequential analysis. J Shoulder Elbow Surg.

[12] Steinmuller L, Bartlomiejczyk S, Fernandez A, Hemmer J, Galois L (2021). Outpatient surgery of the first ray of the foot: post-operative pain monitoring at home. Foot Edinb Scotl.

[13] Aubrun F, Chrétien E, Letrilliart L, Ginoux M, Belhassen M, Lanteri-Minet M (2017). What are the therapeutic alternatives to dextropropoxyphene in France? A prescribers´ survey. Anaesth Crit Care Pain Med.

[14] SoSci der onlineFrigeborgen SoSci Survey - the Solution for Professional Online Questionnaires.

[15] Cleveland Clinic Sample Size Estimation in Clinical Research: from Randomized Controlled Trials to Observational Studies.

[16] Heel RC, Brogden RN, Pakes GE, Speight TM, Avery GS (1980). Nefopam: a review of its pharmacological properties and therapeutic efficacy. Drugs.

[17] Moore RA, Derry S, Aldington D, Wiffen PJ (2015). Single dose oral analgesics for acute postoperative pain in adults - an overview of Cochrane reviews. Cochrane Database Syst Rev.

[18] Aubrun F Post-operative Analgesic Effect of Oral Nefopam: a Randomized Controlled Trial (NefPO). Clinicaltrials.gov.

[19] Aymard G, Warot D, Demolis P, Giudicelli JF, Lechat P, Le Guern ME (2003). Comparative Pharmacokinetics and Pharmacodynamics of Intravenous and Oral Nefopam in Healthy Volunteers. Pharmacol Toxicol.

[20] Chawla J, Le Guern M-E, Alquier C, Kalhorn TF, Levy RH (2003). Effect of route of administration on the pharmacokinetic behavior of enantiomers of nefopam and desmethylnefopam. Ther Drug Monit.

[21] Riviere JP Retrait en 2011 du dextropropoxyphène: quel impact sur l'utilisation des autres antalgiques?.

[22] Van Ganse E, Belhassen M, Ginoux M, Chrétien E, Cornu C, Ecoffey C (2018). Use of analgesics in France, following dextropropoxyphene withdrawal. BMC Health Serv Res.

[23] Cabañero D, Maldonado R (2021). Synergism between oral paracetamol and nefopam in a murine model of postoperative pain. Eur J Pain.

[24] National Library of Medicine (U.S.) (2020). Nefopam/Paracetamol Fixed Dose Combination in Acute Pain After Impacted Third Molar Extraction. Clinicaltrials.gov; Identifier NCT04622735 [Internet].

[25] Durrieu G, Olivier P, Bagheri H, Montastruc JL, French Network of Pharmacovigilance Centers (2007). Overview of adverse reactions to nefopam: an analysis of the French Pharmacovigilance database. Fundam Clin Pharmacol.

[26] Revol B, Delorme J, Jouanjus É, Spadari M, Djezzar S, Lepelley M (2021). Trente ans d´abus de néfopam en France. Therapie.

[27] Joo YC, Ko ES, Cho JG, Ok YM, Jung GY, Kim KH (2014). Intravenous Nefopam Reduces Postherpetic Neuralgia during the Titration of Oral Medications. Korean J Pain.

[28] Richards BL, Whittle SL, Buchbinder R (2012). Neuromodulators for pain management in rheumatoid arthritis. Cochrane Database Syst Rev.

[29] Smith BH, Hardman JD, Stein A, Colvin L, SIGN Chronic Pain Guideline Development Group (2014). Managing chronic pain in the non-specialist setting: a new SIGN guideline. Br J Gen Pract J R Coll Gen Pract.

[30] Gerbershagen HJ, Aduckathil S, van Wijck AJM, Peelen LM, Kalkman CJ, Meissner W (2013). Pain Intensity on the First Day after Surgery. Anesthesiology.

[31] Girard P, Chauvin M, Verleye M (2016). Nefopam analgesia and its role in multimodal analgesia: A review of preclinical and clinical studies. Clin Exp Pharmacol Physiol.

[32] Visser PS, Krosnick JA, Marquette J, Curtin M (1996). Mail Surveys for Election Forecasting? An Evaluation of the Columbus Dispatch Poll. Public Opin Q.

